# A genealogy of epidemiological reason: Saving lives, social surveys and global population

**DOI:** 10.1057/s41292-017-0055-2

**Published:** 2017-07-03

**Authors:** David Reubi

**Affiliations:** Department of Social Science, Health & Medicine, King's College London, East Wing Building, Strand, London W2R 2LS, UK

**Keywords:** epidemiology, saving lives, metrics, biopolitics, social survey, global health

## Abstract

Metrics have become all pervasive in global health today. Instead of highlighting their advantages or shortcomings, this article builds on Hacking's notion of historical ontology and explores their political, conceptual and material conditions of possibility. Drawing on research on the Bloomberg Initiative to Reduce Tobacco Use in Developing Countries, one of the largest international efforts to address the non-communicable disease epidemic in the global South, the article starts by introducing the notion of epidemiological reason – a thought style associated with modern epidemiology that undergirds the metrics permeating the global health field and which is made of a multiplicity of elements, from the ethical imperative to save lives to the social-scientific technique of the survey and the concept of global population. The article then goes on to explore the genealogy of this thought style, arguing that three epistemological ruptures have been critical to its development: the reconfiguration of power articulated around a biopolitics of population in the eighteenth and nineteenth centuries; the twentieth-century shift in medical thought marked by the emergence of surveillance medicine and the idea of lifestyle; and the re-organisation of world health informed by globalisation theories at the start of the twenty-first century.

## Introduction

In the Spring of 2007, *The Lancet,* arguably the most influential journal in the field of global health, published a piece entitled: “How to Prevent 100 Million Deaths from Tobacco”. In it, the authors – Michael Bloomberg, the billionaire entrepreneur and then Mayor of New York City, and Tom Frieden, the City's then Health Commissioner and now Director of the US Centres for Diseases Control (CDC) – announced the launch of one of the first, major global health initiatives to tackle the growing non-communicable disease (NCD) epidemic in the global South: the Bloomberg Initiative to Reduce Tobacco Use in Developing Countries. Financed by the Bloomberg and Gates foundations and building on New York's successful anti-smoking campaign, the Initiative brings together international and American health organisations with local government agencies and civil society groups to fight smoking in low- and middle-income countries (Bloomberg Philanthropies, 2011). One of the Initiative's most striking features is how numbers and quantification practices permeate its rhetoric, organisation and management. In their *Lancet* article, for example, Frieden and Bloomberg (2007, p. 1758) justify the Initiative's focus on tobacco by emphasising that it is “the world's leading agent of death” and will “kill one billion people prematurely during this century”. They also explain that the Initiative is built around a “quantifiable target” – a decline in 'global adult smoking prevalence' of “20 per cent by 2020” – and a “package” of measures whose “effectiveness” has been evidenced through rigorous evaluations (ibid, pp. 1758 and 1761). Likewise, they point out to the Initiative's *ad hoc* epidemiological surveillance system that allows to “monitor results” and “guide programme implementation” (ibid, p. 1760). This central role of numbers and quantification practices is, of course, not unique to the Bloomberg Initiative. Indeed, it is common to the entire global health field (Adams, 2016a; Gorsky and Sirrs, 2017; Mahajan, 2017) and part of a wider “quantificational spirit” that shapes the way in which most aspects of political, economic and social life are administered nowadays (Power, 2004, p. 766; e.g., Power, 1999; Shore and Wright, 2015) – a spirit that will only become more prevalent in the coming years as we enter “the era of big data” (Boyd and Crawford, 2012, p. 663).

Scholarly work on quantification tends to be strongly polarised and the literature on global health metrics is no exception in that respect (Power, 2004). On one side, we have health experts and epidemiologists who, for the most part, celebrate the enlightening power of numbers, which they see as a product of scientific progress and the triumph of reason over ideology and tradition. For them, metrics make health issues visible, identify effective public health strategies and determine the most rational way to organise and manage healthcare (e.g., Murray and Evans, 2003; Jamison et al., 2006; Lopez et al., 2006; Horton, 2013). On the other side, there are critically minded scholars in the social sciences and humanities for whom numbers and their association with “dangerous neoliberal arrangements” and “forms of profit seeking” need to be denounced (Adams, 2016b, pp. 189–190). For these scholars, metrics will always fail to convey the complexities of people's everyday lives and foreclose other, non-quantitative forms of politics and healthcare (e.g., Birn, 2009; Erikson, 2012; Storeng and Behague, 2014; Adams, 2016a). In contrast to this literature and its concerns with the benefits and limitations of quantification, the present article seeks to understand how metrics came to dominate the field of global health in the first place. Specifically, focusing on the Bloomberg Initiative and building on Ian Hacking's (2002) notion of historical ontology and his work on the history of statistical reason (Hacking, 1990, 1992; cf. also: Porter, 1995; Desrosières, 1998), this article queries the conditions of possibility of contemporary global health metrics and explores the ways in which the forms of expertise, institutions and practices that undergird these metrics have been assembled over time. To do so, I start by giving a brief account of the Bloomberg Initiative before introducing the notion of epidemiological reason to capture the thought style or logic that weaves together and shapes the metrics permeating the Initiative. I then explore the genealogy of this thought style and argue that three successive epistemological ruptures were critical to its articulation. I conclude by reflecting on the insights that this genealogical approach offers to the study of global health metrics.

## The Bloomberg Initiative and Epidemiological Reason

The Bloomberg Initiative to Reduce Tobacco Use in Developing Countries was launched by Michael Bloomberg through his charity, Bloomberg Philanthropies, in 2007 (Bloomberg Philanthropies, 2011). One year later, he was joined by Bill and Melinda Gates, whose foundation added another US$ 125 million to the nearly US$ 1 billion invested by Bloomberg (Gates Foundation, 2015; Bloomberg Philanthropies, 2016). For Bloomberg and the Gateses, the Initiative is part of their wider commitment to “make a better world” and “giving back” to the community (Bloomberg, 2001, p. 242; Gates and Gates, 2010, p. 2). As already mentioned, the Initiative builds on the anti-smoking campaign that Health Commissioner Tom Frieden and his team ran in New York City during Bloomberg's mayoralty. For Bloomberg and Frieden, who had been tasked by the Mayor with designing the Initiative, the latter was a great opportunity to showcase their work in New York at a time when, following the adoption of the WHO Framework Convention on Tobacco Control (FCTC), there was growing momentum in the international fight against smoking (Reubi and Berridge, 2016). In keeping with Frieden's original blueprint, the Initiative is organised around a small number of official partners: the WHO's Tobacco Free Initiative, the CDC, the Johns Hopkins' School of Public Health and a few, mostly American charities like the Campaign for Tobacco Free Kids. The mandate of these partners is to help reduce tobacco use in the global South by building tobacco control capacity on the ground, setting up anti-smoking coalitions, establishing epidemiological surveillance systems and supporting local NGOs and government agencies to pass anti-smoking laws, raise tobacco taxes, run public awareness campaigns and so on (WHO, 2013).

As outlined in the introduction, the Bloomberg Initiative is saturated with metrics. These metrics, I suggest here, are part of a wider thought style – epidemiological reason – at work throughout the Initiative. Broadly speaking and before examining it in more detail below, I conceive this style of reasoning as a grid of intelligibility and action articulated around a series of ethical values, management principles, quantification techniques and political categories, some of which are closely related to modern epidemiology. This understanding draws, of course, on the work of Hacking (1992, 2002) and others (e.g., Fleck, 1979; Hass, 1992; Mirowski and Plehwe, 2009). According to this more or less coherent body of work, a thought style is similar to Michel Foucault's episteme or discursive formation (Rose, 2000, p. 5; Hacking, 2002, p. 180). It is a “way of seeing and practising” (Rose, 2000, p. 5), a “set of epistemic commitments” (Mirowski and Plehwe, 2009, p. 417) that “makes possible certain ideas and renders others unthinkable” (Hacking, 2002, p. 180). A style of reasoning is often associated, though by no means coterminous, with a formalised body of knowledge like economics, psychiatry or – as is the case here – modern epidemiology (Hacking, 1992; Rose, 2000). This association does not mean, however, that the influence of thought styles is constrained to the production of scientific truth. On the contrary, they often spill over and shape political, economic and social life (Hass, 1992; Mirowski and Plehwe, 2009).

At the heart of the epidemiological thought style that informs the Bloomberg Initiative is the desire to save and improve the greatest number of human lives. This desire to save lives is expressed repeatedly in the Initiative's official materials. So, for example, a pamphlet published by the Gates foundation states that:

[We] work to help all people lead healthy, productive lives … [Our tobacco control work is about] making a difference and saving lives (Gates Foundation, 2009, p. 1).

Similarly, Michael Bloomberg, in a speech made to activists working for the Initiative, mused that:

[The Initiative] is a great opportunity to have to a positive impact on the world … We can take great pride in helping people and their children have better, longer and healthier lives.

Also at the heart of epidemiological reason is the belief that efforts to save lives have to be based on rigorous epidemiological data in order to be effective. A good illustration is the way experts working for Bloomberg and Gates carefully analysed existing global health statistics before deciding to focus their efforts on tobacco. Four epidemiological research and surveillance projects, which all established that smoking was now the leading cause of death worldwide, were especially influential in that respect: Chris Murray and Alan Lopez' Global Burden of Disease venture; Richard Peto's work on global, smoking-attributable mortality; the Tobacco Atlas project based on the American Cancer Society's NATIONS monitoring system; and the CDC's Global Youth Tobacco Survey (GYTS) (e.g., Lopez et al., 2006; Peto and Lopez, 2000; Mackay and Eriksen, 2002; Warren et al., 2009). As two public health specialists involved in setting up the Initiative remembered:

The decision to focus on tobacco came entirely from the data... We were looking for an area related to a very large number of deaths worldwide... So, we looked at the numbers and analysed them. They showed that smoking was now the world's leading single cause of death with over six million deaths per year compared with three for AIDS, two for tuberculosis, one for malaria … We knew our focus had to be tobacco.

Bill Gates was convinced by the numbers. He has a very mathematical mind. He just read all the information. He read the Global Burden of Disease. He read the Tobacco Atlas … And he did the maths. He saw the number of lives that can be saved. That is why he became interested in tobacco and teamed up with Bloomberg.

Another good example of this belief that efforts to save lives need to be based on rigorous data to be effective is the importance that the Initiative attaches to epidemiological surveillance. As already mentioned, the Initiative has its own surveillance system: the Global Adult Tobacco Survey (GATS), which is run by the CDC in collaboration with the WHO and national health ministries and statistical agencies. Unlike its predecessor, the GYTS, which is a school-based survey of 13–15 year olds, the GATS is a household survey of adults that monitors tobacco use and key tobacco control indicators like public attitudes towards smoking (CDC, 2009). For the experts at Bloomberg and Gates, this surveillance system is deemed critical to the effective management of the Initiative and to determining whether it is achieving its goal to reduce “global adult smoking prevalence” below “20 per cent by 2020" (Frieden and Bloomberg, 2007, p. 1761). As one of them argued:

On-going, systematic surveillance is a key public health tool. It guides programme implementation. It makes it possible to monitor results and evaluate whether interventions are effective … Surveillance provides critical information … Who smokes? Where? How much? Who is doing what in terms of policy? … If you cannot measure it, you cannot manage it.

Within the epidemiological thought style at work throughout the Initiative, the notion of epidemiological data is closely associated with a very specific quantification practice: social surveys that focus on unhealthy lifestyles like smoking. This is evident when looking at the epidemiological research and surveillance projects that inform the Bloomberg Initiative, from the Global Burden of Disease venture and Peto's studies on global smoking-attributable mortality to the American Cancer Society's NATIONS project and the CDC's GYTS and GATS. First, all these projects are concerned with quantifying the diverse dimensions of tobacco use. For example, the Global Burden of Disease venture and the research done by Peto and his colleagues both aim to measure tobacco consumption and its impact on disease, disability and death (Peto et al., 1994; Murray and Lopez, 1996; Ng et al., 2014). Similarly, the NATIONS, GYTS and GATS systems seek to quantify some of the behavioural, economic, social and political aspects of smoking, including tobacco consumption and second-hand smoke exposure; the production, trade and price of cigarettes; people's beliefs about tobacco; and the number of anti-smoking laws adopted (Shafey et al., 2003; Warren et al., 2009). Second, all these projects also use one or another type of social survey. So, the GYTS and GATS are, respectively, built around standardised school-based and household surveys carried out by the CDC and its partners (Warren et al., 2009). Similarly, Peto and his colleagues' work is built around a few large observational studies, which they conducted in the USA, India and China and for which they interviewed people about their smoking habits and medical history (WHO, 1990; Peto et al., 1994; Liu et al., 1998). Last but not least, the NATIONS system and the Global Burden of Disease project operate by collecting and integrating surveys that have already been conducted by other agencies, like the Demographic and Health Surveys funded by USAID and the morbidity surveys carried out by community-based hospitals (Shafey et al., 2003; Smith, 2015).

Data within epidemiological reason is also – both in terms of its production and use – closely associated with experts in epidemiology and the specialised public health institutions in which they train and work. There is a myriad of such experts and institutions involved in the Initiative. To start with, many epidemiologists and other public health specialists with close links to the CDC – America's premier federal public health agency – hold or have held high-level positions within the Bloomberg and Gates foundations that involve commissioning, consulting and drawing upon epidemiological data to set up and/or manage the Initiative. Tom Frieden and Kelly Henning are cases in point. Frieden, who played a critical role in designing the Initiative, is a medical doctor and former CDC Epidemiological Intelligence Service Officer. Similarly, Henning, the Director of International Health Programmes at Bloomberg Philanthropies and, as such, the head of the Initiative, is a physician trained in epidemiology at the CDC. David Fleming and Kathy Cahill, who together devised and managed the Gates Foundation's tobacco control strategy, are another two good examples. Fleming, also a physician by training, worked as Oregon's State Epidemiologist and CDC's Deputy Director of Public Health Science before joining the Gates Foundation. Likewise, Cahill trained in public health and was CDC's Director for Planning, Policy and Evaluation before being hired by the Gates Foundation. Furthermore, there are also many epidemiologists working for public health schools or government health agencies who, while not directly employed by the Initiative, make critical contributions to its operation by providing the epidemiological data it needs. So, for example, Samira Asma and her group of researchers at the CDC have been mandated by the Bloomberg and Gates foundations to manage the GATS. In the same way, Gates funds Chris Murray, Alan Lopez and their team of epidemiologists at the Institute of Health Metrics and Evaluation (IHME), University of Washington, to run the Global Burden of Disease project (Smith, 2015). A further example is Oxford epidemiologist Richard Peto who, together with colleagues like Prakash Gupta in Mumbai and Bo-Qi Liu in Beijing, has produced much of the global smoking-attributable mortality data on which the Initiative is based (WHO, 1990; Liu et al., 1998).

Last but not least, the epidemiological logic at work within the Initiative is also characterised by a focus on the global and the notion of global population. In official documents and reports, the Initiative is repeatedly depicted as a global effort to address a worldwide threat. So, for example, in his writings, Frieden describes the joint Bloomberg-Gates anti-smoking efforts as a “global initiative” to combat “the world's leading preventable epidemic” (Frieden, 2007, slide 24; Frieden and Bloomberg, 2007, p. 1761). In the same way, the Gates Foundation (2009, 2015) explains that the Initiative is an international “effort to combat the global tobacco epidemic” and “achieve a smoke-free world''. The assertion that tobacco use is a global threat is based on the epidemiological research and surveillance projects discussed above. Indeed, the aim of these projects is to quantify tobacco use and its burden for what is termed “the global population” (Lopez et al., p. 8; CDC, 2015, p. 32). To illustrate, the aim of the GYTS and GATS is, according to the CDC, to “monitor worldwide tobacco use” (Warren et al., 2009, p. 7). Likewise, Lopez and his colleagues describe the goal of the Global Burden of Disease as being the “quantification of the impact of diseases, injuries and risk factors on the population health globally” (Lopez et al., 2006, p. 5). To achieve that goal, these projects build on transnational collaborations between public health schools, international organisations, philanthropies, charities and government agencies to run social surveys across the globe and, where data is inexistent, generate estimates using sophisticated modelling practices (Peto et al., 1994; Smith, 2015). Much of the data from these projects comes in the form of metrics that seek to capture every aspect of global tobacco use, from the “global adult smoking prevalence” to the “worldwide mortality from tobacco" (Peto and Lopez, 2000, p. 4; Frieden and Bloomberg, 2007, p. 1761). An extract from the Tobacco Atlas offers a striking example:

Almost 1 billion men in the world smoke … Almost 250 million women in the world are daily smokers … Over 15 billion cigarettes are smoked worldwide everyday … [There are] 4.2 million premature deaths from tobacco worldwide … Over five trillion cigarettes are manufactured [globally] (Mackay and Eriksen, 2002, pp. 24–48).

Often, these metrics are further broken down by geo-political region or country and displayed in tables and world maps (e.g., Mackay and Eriksen, 2002; Lopez et al., 2006; CDC, 2015). As experts working for the Initiative explain, these metrics, tables and maps “help us see the epidemic in its global scale” and “sell the need for worldwide tobacco control” (Warren et al., 2009, p. 7).

## A Genealogy

In contrast to much of the scholarly work on quantification in global health, my aim here is not to celebrate the power of epidemiological reason or expose its shortcomings. Instead, my concern is to understand how this thought style has come into being. Put differently, my objective is to chart the way in which the central tenets of epidemiological reason – the will to save lives, the belief in the importance of data, social surveys, epidemiologists and global population – were articulated and assembled over time. Specifically, I explore below three epistemological breaks that, I suggest, were critical in the making of this thought style: the reconfiguration of political power around the idea of population in the long eighteenth and nineteenth centuries; the shift from pathological anatomy to surveillance medicine in the twentieth century; and the passage from international to global health at the start of the twenty-first century.^1^ To espouse such a line of inquiry is to build on Hacking's (2002) historical ontology and, in particular, on his and others' research on the making of statistical reason (e.g., Hacking, 1990, 1992; Porter, 1995; Desrosières, 1998). Put simply, historical ontology is a kind of genealogy in the sense Michel Foucault gave to the expression (Hacking, 2002, chapter 1). In other words, it is a form of critique that, eschewing both celebration and denunciation, seeks to unsettle the “feeling of inevitability” we attach to many of the “things, classifications, ideas, kinds of people and institutions” that make up our current ways of being and thinking (Hacking, 2002, pp. 5, 21). To do so, it “emphases the contingency of the predicaments we find pressing or inescapable” today by tracing the “specific, local historical ways” and “epistemological ruptures” that have made them possible (Hacking, 2002, pp. 3, 24, 93).^2^

### Faith in Numbers and the Biopolitics of Population

Some key aspects of epidemiological reason can be traced back to the rupture in political thought that took place in Europe and North America towards the end of the early modern period. As Foucault (1998, 2009, 2014) argued, the West experienced at the time a significant reformulation of the mechanisms of power around the new notion of population. In the medieval tradition, a good government was part of the natural “world order willed by God” (Foucault, 2009, p. 349). As a consequence, “the art of government” in the Middle-Ages was about “knowing the positive laws of the country, the natural laws imposed on all men and, of course, the laws and commandments of God" (Foucault, 2009, p. 273). As societies in Europe and North America modernised, there was a radical departure from this old cosmological–theological framework. An initial break was made in theories of *raison d'Etat* and mercantilist philosophies that dominated political thought in the long eighteenth century (Lemke, 2010). The art of government, according to these theories and philosophies, was not about “the cosmos, nature or the divine” anymore but about “the preservation, expansion and felicity of the state" (Foucault, 2009, p. 257). It is as part of this new understanding of government that the notion of population, viewed as a measure of the state's power where strong states have large populations, was first sketched. A further break from the medieval tradition was made in political economy, a body of thought that become influential from the mid-1700s onwards (Lemke, 2010). For political economists, the population was the final end and object of government, rather than simply an indicator of the state's power. Government, according to them, was about improving the condition of the population understood as “an ensemble of individuals having between themselves relations of coexistence and constituting therefore a specific reality” (Foucault, 2014, p. 117).

A crucial aspect of this new form of power was the way it redefined the relationship between government and life. In the medieval tradition, this relationship was characterised by 'deduction': the sovereign had a right to dispose of the lives of its subjects in the same way it had a right to seize their goods and services (Foucault, 1998, p. 136). In contrast, the relationship, in mercantilist and political economic philosophies, was marked by a will to secure, foster and augment life. Indeed, for these theories, the population was not only a political and economic reality but also a biological one. Therefore, in order to improve the condition of the population, tending to the body politic and economic was not sufficient; it was also critical to attend to the species body. For Foucault (1998, pp. 139–143), the recognition of the “biological existence of a population” and the will to augment it through what he called a “biopolitics of population” was the hallmark of 'modernity' and has characterised modern Western societies up to this day (Lemke, 2010). In the long eighteenth and nineteenth centuries, biopolitical efforts to secure and augment the life of the population took the form of public hygiene and sanitation (Desrosières, 1998). For the most part, public hygiene and sanitation was concerned with the deleterious urban environment brought about by industrialisation and advocated public health policies that drew on anticonta-gionist theories of miasma, such as sewers, waste collection and the ventilation of houses.

Modern biopolitical efforts to foster life came together with the development of new quantification practices to measure the birth, marriage and death rates – or vital statistics – of populations (Szreter, 1991; Desrosières, 1998). These practices, whose articulation in nineteenth-century Europe and North America represented a significant moment in the history of statistical reasoning, were centred around two specific techniques: (1) vital registration systems to record births, marriages and deaths and (2) censuses to enumerate each individual in the population. Administered by public health reformers with an interest in statistics like Englishman William Farr and newly established national statistics agencies like the UK's General Register Office (GRO), these techniques involved national networks of civil registry offices, standardised death certificates, electric cross-tabulation machines and causes-of-death classifications (Szreter, 1991; Desrosières, 1998). In a modern regime of power, the knowledge about the population's vital statistics generated through these practices was deemed essential to a good politics of health. Indeed, in contrast to the medieval tradition, where the prince governed in accordance to a natural or divine order, the modern art of government was based on truth and, especially, scientific truth (Foucault, 2009, pp. 350–351). Moreover, the growing role of quantitative expertise and trust in numbers in the government of public life during this period meant that more often than not scientific truth was numerical (Hacking, 1990; Porter, 1995). It is therefore unsurprising that the birth and death rates calculated by the new experts in vital statistics were quickly deemed essential in guiding public hygiene and sanitation policies. A good example is the way in which the statistics computed by the GRO under William Farr were received and used in the UK during the nineteenth century (Szreter, 1991). Through its regular statistical bulletins, the GRO did more than just provide authoritative information on death rates. It also actively campaigned for districts throughout the country to adopt environmentalist public health policies by raising awareness about the amount of preventable deaths and setting acceptable death rate targets for local authorities to achieve.

What is important to note in relation to the genealogy mapped out here is that, at the start of the twentieth century, a couple of key tenets of the epidemiological thought style at work throughout the Bloomberg Initiative were in place in most nation-states across Europe and North America. First, the desire to save and improve the greatest number of human lives that informs the Initiative is a consequence of the reconfiguration of political thought around population and the transformation of the relationship between power and life at the end of the early modern period. Specifically, this desire grows out of the recognition of the biological dimension of human existence and the will to augment it through a biopolitics of population first expressed in mercantilist and political economic theories and characteristic of most modern Western societies ever since (Foucault, 1998). Second, the belief held by experts working for the Initiative that rigorous epidemiological data is critical for safeguarding human lives effectively is the result of the history of statistical reasoning and the central role that scientific knowledge and numbers came to play in regimes of power in the modern era (Hacking, 1990; Porter, 1995). Precisely, this belief follows directly from the view, embodied in the work of William Farr and the GRO in nineteenth-century UK, that knowing the population's vital statistics was essential to good public hygiene and sanitation policies (Desrosières, 1998).

But, while these two central features of epidemiological reason were in place by the early twentieth century, many other key tenets remained absent. One of these was the quantification practices centred on the social survey and concerned with measuring the many dimensions of tobacco use. As mentioned, the rupture in political thought at the end of the early modern period had seen the development of another kind of quantification practices, which were built around vital registration systems and geared to calculate the population's birth and death rates. While these vital statistics systems set up during the nineteenth century shared some institutional and technical features with the survey-centred quantification practices characteristic of the Bloomberg Initiative, their aim was very different. Indeed, they purported to measure the effects of the environment – and, in particular, the insalubrious urban milieu of mass industrialisation – on human life by counting the number of deaths. They did not conceptualise and could not measure an unhealthy individual behaviour like smoking and its various biological, social, political and economic dimensions. To quantify tobacco use in the way epidemiologists associated with the Bloomberg Initiative do today, one would have to wait for the emergence of surveillance medicine and the development of the social survey in the mid-twentieth century. Another important feature of epidemiological reason that was also absent in the early 1900s is the idea of a global population. It is true that, as we discussed above, the notion of population was articulated as early as the 1700s and 1800s by mercantilists and political economists (Foucault, 2009). But theirs was a national population, not a global one. It was the population of the nation-state that was being articulated in Europe and America from the late eighteenth century onwards (Anderson, 1983). As we will see, the idea of a global population would only emerge in the late twentieth century as part of the shift from international to global health.

### Modern epidemiology and the social survey

As Armstrong (1983, 1995; cf. also Weisz, 2014) has argued, the mid-twentieth century saw a rupture in medical thinking, with the emergence of surveillance medicine and the displacement of pathological anatomy. A critical aspect of this rupture was a shift in the medical gaze from the 'pathological lesion' located in the diseased body of the patient to the 'risk factors' of possible future diseases for everyone in the community, both the ill and the seemingly healthy. The notion of risk factor was a product of actuarial studies in the life insurance industry and research on child hygiene in the early twentieth century (Rothstein, 2003; Armstrong, 2009). Its rise to pre-eminence was also associated with a concomitant change in North American and European disease patterns, which saw infectious diseases drop while chronic conditions like coronary heart disease and lung cancer increased markedly and began to be framed as a medical issue rather than the endpoint of natural ageing (Armstrong, 2014). The new focus on risk factors had two consequences for medical thought. First, the concern with future disease potential rather than actually existing diseases led to the “dissolution of the clinical categories of healthy and ill” and a “problematisation of the normal” (Armstrong, 1995, p. 395). Second, with the search for causes of future illness now stretching from physiological attributes (e.g., blood pressure, weight) to behavioural patterns or lifestyles (e.g., diet, smoking), medical thought was pushed beyond the body of pathological anatomy and into an increasingly “extracorporeal space” (ibid, p. 401).

This shift in medicine's gaze came together with changes to both its modes of knowing and its forms of care. While the pathological lesion was investigated through clinical examinations, laboratory tests and post-mortems with the ambition of discovering specific, biological causal mechanisms, risk factors were charted through social surveys with the aspiration of establishing statistical correlations (Brandt, 1990; Armstrong, 1995). The investigative techniques and theories of causality associated with research on risk factors were the products of modern epidemiology. Up to the 1920s, epidemiologists had been physicians with no specific training often working as public health officers in government. Their main duty was to investigate the sanitary conditions that caused infectious disease outbreaks, using field investigations and laboratory tests (Amsterdamska, 2005). This changed after the 1930s. Universities and medical schools set up chairs and degrees in epidemiology, with the latter developing into a recognised, modern academic discipline weaving together elements from statistics, medicine and sociology and informed by the ideals of welfarism (Armstrong, 1983; Susser and Stein, 2009). For the experts in this new academic discipline, the task was to survey and alter the “harmful ways of living” that were responsible for the mounting “modern epidemic” of chronic diseases (Morris, 1955, pp. 396 and 401; cf. also Oppenheimer, 2006). As with modes of knowing, the rupture in medical thought also brought about the reconfiguration of forms of care. While treatment in pathological anatomy was limited to the bodies of the sick in hospital beds, care in surveillance medicine was ambulatory and on-going and delivered through community health centres. It also assumed responsible patients who would actively engage in their education and treatment, which comprised health promotion campaigns, screening tests and life-long drug regimens (Armstrong, 1995; Petersen and Lupton, 2000). This shift in forms of care was closely related to both the postwar articulation of the welfare state and its politics of social solidarity and the concurrent development of a 'new' public health that, unlike the 'old' public hygiene and its focus on the environment, was concerned with lifestyles (Armstrong, 1983).

Importantly for us, the rise of surveillance medicine brought a change in how the life of the population was conceived and quantified. Specifically, there was a move from the old vital statistics machinery, which calculated the impact of insalubrious environments on human mortality, to the new technique of the social survey, which measured the biological, social, economic and political aspects of unhealthy lifestyles (Armstrong, 1983; Brandt, 1990; Oppenheimer, 2006). As mentioned, the social survey grew out of the matrix of modern epidemiology in the mid-twentieth century. The experts in this new academic discipline developed and used two major types of survey in particular. The first was the morbidity or health survey (Weisz, 2014). Carried out by health authorities to guide the provision of healthcare, this type of survey collects information about the population's health status and behaviours as well as its healthcare access, utilisation and expenditure. It does so through sociological analyses of people's attitudes and experiences of disease like health profile questionnaires and subjective health indexes, sometimes augmented by laboratory and clinical examinations. A good example is the US National Health Interview Survey (NHIS) – an interview-based household survey about the diseases, disabilities, lifestyles, hospital visits and healthcare expenditure among Americans, carried out regularly by the US National Centre for Health Statistics (NCHS) since the late 1950s (CDC, 2010). The second type of survey was the observational study (Oppenheimer, 2006; Susser and Stein, 2009). Run by epidemiologists working in universities and medical schools, this type of survey aims to measure the impact of possible risk factors on the development of a given chronic disease, using sociological and clinical examinations in combination with statistical techniques like multivariate analysis and control groups. A well-known illustration is the research carried out by Jerry Morris at the London School of Hygiene and Tropical Medicine (LSHTM) in the 1950s. Drawing on a mix of direct observations, interviews, clinical examinations and autopsies, Morris showed how physical inactivity was a key risk factor for cardiovascular disease by comparing London bus drivers, who sat behind their wheel all day and had a high risk of heart attacks, with bus conductors, who walked up and down the bus checking tickets and rarely suffered heart attacks (Weisz, 2014).

Interestingly, smoking features centrally in the story of modern epidemiology and the social survey (Brandt, 1990). To begin with, research on the causes of lung cancer and, particularly, the work of British statistician Austin Bradford Hill and physician Richard Doll at the LSHTM was critical in developing the observational study as a recognised research design. Hill, Doll and the LSHTM embodied the mixture of statistical, medical and sociological expertise and the ideals of welfarism and social solidarity that were so typical of modern epidemiology, with Doll in particular exhibiting a strong social conscience that led him to join the Socialist Medical Association and advocate for the British National Health Service (Armstrong, 1983; Weisz, 2014). Alerted by the growing mortality statistics for lung cancer computed by the GRO, Hill and Doll ran two of the first, large population studies to determine the risk factors associated with lung cancer: a case–control study comparing patients suffering from lung cancer with patients having other diseases using interviews with patients, analyses of medical records and autopsies; and, later, a cohort study comparing smoking and non-smoking British doctors using questionnaires and death certificates (Doll and Hill, 1950, 1954). In addition to demonstrating that lung cancer was caused by individual behaviours like smoking rather than, as it was then thought, environmental factors like pollution, their studies also pioneered a research design that would become the “paradigm for modern epidemiology” (Boyle, 2005). Moreover, as a direct consequence of the work of Hill and Doll, questions about smoking became increasingly prominent in health surveys. The USA, where an increasing number of nation-wide social surveys on smoking-related behaviours were carried out from the 1960s onwards, provides a good illustration (CDC, 2014). At first, questions about cigarette use, age of initiation and brand preference were integrated into general health surveys like the NHIS mentioned above. Later, smoking became one of the main subjects for interview-based surveys focusing on health-related risk behaviours like the Behavioural Risk Factors Surveillance System (BRFS) run by the CDC. More recently, one has seen the development of interview-based surveys focusing solely on smoking, like the CDC's National Youth Tobacco Survey (NYTS) and National Adult Tobacco Survey (NATS), which explore topics like tobacco use and attitudes to smoking (CDC, 2014).

Again, what is critical here is how the mid-twentieth-century advent of surveillance medicine set in place a further two key features of contemporary epidemiological reason. The first of these features are the experts in epidemiology and specialised public health institutions that, because of their ability to produce, interpret and use data, are so critical to the management of the Bloomberg Initiative. These experts and institutions have been made possible by the development of a modern science of epidemiology concerned with chronic diseases and lifestyles that accompanied the rise of surveillance medicine. Indeed, most epidemiological experts who have influenced the Initiative, from Tom Frieden to Chris Murray, have been trained in the new discipline of public health and epidemiology developed by socially concerned physicians, statisticians and social scientists in universities and medical schools after the 1930s. Richard Peto, whose research on global, smoking-related mortality has been so influential for the Bloomberg Initiative, is probably the best example. Trained in natural science and statistics, he turned to epidemiology after working closely with Richard Doll, the physician and committed socialist who helped establish the link between tobacco and lung cancer and pioneered observational studies. Similarly, almost all the public health institutions which have been involved with Gates' and Bloomberg's anti-smoking efforts, from the CDC and the American Cancer Society to the IHME and the Johns Hopkins School of Public Health, follow and apply the ideas and techniques of modern epidemiology. The CDC offers a good illustration. Formed after World War Two to fight malaria and other infectious diseases, it was comprehensively restructured under William Foege in the late 1970s to address unhealthy lifestyles and chronic diseases and has since become America's premier public health agency (Etheridge, 1992).

The second key feature of epidemiological reason to grow out of surveillance medicine is the social survey. The centrality of this quantification practice within the Bloomberg Initiative was made possible by the development of modern epidemiology and the shift from vital statistics and their interest in the environment to the social survey and its concern with lifestyles. To start with, this shift in how the life of populations was conceived and measured enabled the research and surveillance projects closely associated with the Initiative, from the CDC's GATS to the IHME's Global Burden of Disease, to focus on a behavioural risk factor like smoking. Furthermore, it also gave those projects the research design, be it in the form of the health survey or the observational study, to quantify the different aspects of this behaviour, from tobacco consumption and the disability and death burden it generated to people's attitudes towards smoking, the trade and price of cigarettes and the policy landscape. A good example is Peto and his colleagues' work on global, smoking-related mortality. The large observational studies in the USA, China and India that have been at the heart of this work were closely modelled on the two UK-based case-control and cohort studies on lung cancer and smoking pioneered by Hill and Doll (WHO, 1990; Peto et al., 1994; Liu et al., 1998). Another good example is the CDC's GYTS and GATS. These surveys were modelled on the NYTS and NATS already carried out by the Atlanta-based organisation and, more generally, drew on the methods and infrastructures developed by the CDC while running most North American health-related behaviour surveys over the last 40 years (CDC, 2014).

### Globalisation, health and population

Most features of present-day epidemiological reason were in place by the end of the twentieth century. One key aspect that was still missing was the notion of global population. As mentioned earlier, the notion of population articulated by eighteenth- and nineteenth-century mercantilists and political economists was a national one associated with the idea of the nation-state that began to dominate political thought during the 1800s (Anderson, 1983; Foucault, 2009). It was this concept of national population that permeated both nineteenth-century vital statistics and twentieth-century social surveys and shaped their efforts to measure the life of the nation (Armstrong, 1983; Desrosières, 1998). As we show below, the idea of global population that characterises contemporary epidemiological reason only emerged with the re-organisation of world health around the concept of globalisation at the turn of the twenty-first century.

The post-Cold War period saw a rupture in the government of world health, with a gradual shift from international to global health (Brown et al., 2006; Gaudillière, 2014). In the international health model, which became predominant after the mid-1800s, nation-states were the locus of biopolitical power and the prime agents for securing health (Weir and Mykhalovskiy, 2010; Rees, 2014). They exercised absolute power on their territories and worked together to address issues arising between them through international coordination mechanisms like the WHO and other United Nations agencies. Of course, international health was, at first, a European and American affair, with the colonial territories of Asia, Africa and the Caribbean governed through Empire and colonial medicine. This changed with the end of Empire and the rise of International Development after World War II, when the ex-colonies become members of the community of nation-states, albeit immature ones in need of assistance from more advanced members. It is this international health model that was eclipsed by the emergence of global health in the early 1990s. At the time, many policy and health experts thought that the world was undergoing important changes (Reubi, 2016). Influenced by theories on globalisation, they believed that trade liberalisation together with a revolution in transport and telecommunications was erasing political and geographical boundaries. This, they feared, brought about new transnational health threats – the rapid propagation of infections via air travel; the spread of unhealthy lifestyles like smoking by multinationals – against which the international health paradigm centred on the nation-state was of little help. For these experts, this inability of the nation-state to deal with these threats was compounded by the wider, neoliberal critique of the state as incompetent and wasteful (Mirowski and Plehwe, 2009). The global health model of government was a response to these perceived weaknesses. It has encouraged new forms of transnational action that redistribute biopolitical power to non-state actors and erode the sovereignty of nation-states. These include international treaties vesting organisations with supra-national powers, as with the 2005 International Health Regulations giving the WHO the right to unilaterally declare public health emergencies (Weir and Mykhalovskiy, 2010). They also involve transnational partnerships between philanthropies, civil society groups, private corporations and governments like the Global Alliance for Vaccines and Immunisation and the Global Fund to Fight AIDS, Tuberculosis and Malaria set up at the start of the new millennium (Rees, 2014).

This shift from international to global health is particularly evident in the organisation and management of worldwide anti-smoking efforts. Up to the 1990s, these efforts were organised around the nation-state and international development (Reubi and Berridge, 2016). The International Union against Cancer's influential Smoking and Cancer Programme, which ran from the mid-1970s to the early 1990s, is a case in point. Its aim was to build tobacco control capacity in developing countries by having North American and European experts go and teach local policymakers, civil servants and doctors how to set up national anti-smoking agencies and draw up national tobacco control policies. This changed at the turn of the twenty-first century, when anti-smoking efforts began to build on the idea of globalisation and forms of action involving non-state actors (Reubi and Berridge, 2016). The FCTC, whose making formed a key part of Gro Harlem Brundtland's attempts to revitalise the WHO under the banner of global health, is a great example (Brown et al., 2006). To start with, Brundtland and her allies explicitly framed the FCTC as the answer to smoking understood as a global threat – a “globalisation of unhealthy lifestyles” caused by a “globalisation of trade and marketing” – against which individual nation-states were powerless (Brundtland, 2000, p. 51). Furthermore, the adoption of the FCTC was made possible by the efforts of a pioneering transnational coalition of civil society organisations (Reubi and Berridge, 2016). Another example is the Bloomberg Initiative itself. Like the FCTC, the Initiative is being officially framed as a global solution – “broadening and accelerating the global tobacco control movement” – to a global threat – “the global tobacco epidemic” (Bloomberg Philanthropies, 2011, p. 11; Gates, 2015). Furthermore, the idea of partnership between state and non-state actors is at the heart of the Initiative. This is manifest in the type of organisations – the Bloomberg and Gates foundations, the WHO, the CDC, health charities, public health schools, health ministries and local NGOs – that make up and work for the Initiative. It is also evident in the Bloomberg and Gates foundations' policy documents, which stress the need for “partnerships” with “government, philanthropy and business” as well as “communities and individuals” from “around the world” (Bloomberg Philanthropies, 2015, p. 5; Gates, 2016).

The shift from international to global health was associated with changes in world health metrics, both in terms of the type of actors involved and how they associated and in terms of the sort of numbers being produced. Within the international health paradigm, nation-states had the sovereign power to collect and publish health statistics about their national population and, before decolonisation, the populations in their colonies (Weir and Mykhalovskiy, 2010). As a result, the statistical reports prepared by international health agencies like the WHO could only contain data that nation-states had computed and published or otherwise released through official channels. This meant that most of these reports did not include any global or regional numbers but only tabulated, country by country, the national statistics that these states had formally released. It also meant that, in a postcolonial world where few newly independent states had any statistical expertise, these reports had no or no reliable data for many countries (Reubi, 2016; Smith, 2015). A good example of this conception of health metrics is the pre-1983 editions of the WHO World Health Statistics – an annual report that continued the League of Nations' Annual Epidemiological Reports and contained information about causes and number of deaths, rates of infectious diseases and healthcare facilities.^3^ The information in these reports was tabulated by country, with no single global or regional numbers. Furthermore, the data used for the reports came exclusively from “official national statistics” “released by the competent authorities of the countries concerned”, such as “annual reports of national health administrations” and “questionnaires filled in by the central statistics offices” (WHO, 1976a, p. 5; 1976b, p. 4). Also, the reports covered very few developing countries, with, for example, the 1976 edition including mortality data for only 2 African and 5 Asian countries, half of which were considered to be of “poor quality” (WHO, 1976a, pp. 6–9). Interestingly, while the World Health Statistics themselves did not contain regular data about tobacco until 2005, the WHO's earliest international statistics on smoking dating from the mid-1980s followed a similar format. Thus, they listed the smoking prevalence for each Member State without giving any global figure and the data for developing countries was considered, by experts in the field, to be widely off the mark (WHO, 1985, Annex 1; Reubi, 2016).

This earlier conception of world health metrics began to change in the late twentieth century, when new transnational epidemiological surveillance and research projects were developed. These projects included most of the epidemiological studies that have been so influential for the Bloomberg Initiative like the Global Burden of Disease venture, Peto's research on global smoking-attributable mortality and the CDC's GYTS. They also included other studies like the WHO MONICA Project, which monitored cardiovascular diseases across four continents, and the USAID Demographic and Health Surveys (DHS), which have surveyed maternal and child health in over 80 countries (Tunstall-Pedoe, 2003; Corsi et al., 2012). In keeping with the new global health paradigm and in contrast to the earlier conception of world health metrics articulated around national authorities, these projects were run by transnational coalitions of state and non-state actors: universities, philanthropies, charities, health ministries, aid agencies and inter-governmental organisations. Likewise, far from being limited to official statistics, these projects drew on a variety of sources (Weir and Mykhalovskiy, 2010). Some, like the DHS and the GYTS, conducted their own surveys in a multiplicity of countries, something that was made possible by the erosion of state sovereignty and the rise of affordable air travel. Others like the Global Burden of Disease project built on innovations in information and communication technology to collect, assess, recalibrate and integrate existing stores of health data from across the world into complex statistical models (Smith, 2015). Moreover, unlike the national focus that typified the earlier model of world health metrics, these new transnational projects were “worldwide monitoring systems” concerned with figures and trends that pertained to the “world population” (WHO, 1989, p. 27; Tunstall-Pedoe, 2003, p. 76). The WHO World Health Statistics reports from 1983 onwards offer a good illustration of this shift in the understanding of world health metrics. To start with and in contrast to earlier editions, the figures contained in these reports were “computed by the WHO” and “not necessarily the official statistics of Member States” (WHO, 2005, p. 13). Indeed, to compute these figures, the WHO followed a complex process, collecting any available data “in addition to national statistics” before assessing their quality, compiling them and estimating any missing element (WHO, 2006, p. 7). Furthermore, while these reports continued to list national statistics for each Member State, they now also offered a “global overview” of the “world health situation' by deploying “global indicators” and world maps (WHO, 1983, p. 7; 1993, p. 19; 2005, p. 7). Importantly for us, smoking prevalence quickly became one of these global indicators. Thus, in its 1986 report, the WHO included a section exploring the “worldwide [smoking] trends' and, specifically, the “world consumption of cigarettes” (WHO, 1986, pp. 16–18). It included a map of the world, with countries shaded in different colours marking different consumption levels, and a graphic comparing cigarette consumption and population numbers in the world and some of its geographical regions (WHO, 1986, Figures 4 and 5). And, from 2005 onwards, the editions of World Health Statistics regularly reported on smoking prevalence using the CDC's GYTS data and giving numbers for the globe, the WHO regions and each Member State (WHO, 2005).

To finish, it is worth noting the significant role played by philanthropies in the shift from international to global health and the associated changes in world health statistics (Reubi, 2017). Of course, the involvement of philanthropies in world health and metrics is not new. The Rockefeller Foundation, for example, sought to spread vital statistics to the mostly British colonies of South-East Asia in the first half of the twentieth century, funding the League of Nations' weekly radio-telegraphic epidemiological bulletins drafted by its Far Eastern Bureau in Singapore (Bashford, 2006). However, the recent shift to global health has led to the re-emergence of philanthropies as major actors in world health (Rushton and Williams, 2011). Indeed, as alluded to, policy and health experts have increasingly come to view philanthropic foundations, usually in partnership with other non-state actors, as the answer to the perceived weaknesses of the international health model centred on the nation-state. One area in which philanthropists active in world health have been particularly keen to invest is the development of new global health metrics, with many of them funding transnational surveillance systems and epidemiological research projects. Gates and Bloomberg's financial support of the IHME's Global Burden of Disease project and the CDC's GATS – which are critical to contemporary epidemiological reason – are cases in point. These philanthropists' interest in metrics grows, at least in part, out of the practices of audit that came to dominate the government of corporate and public life in the late twentieth century (Power, 1999; Shore and Wright, 2015). Gates, for example, is usually regarded as “one of the high priests” of the new culture of audit “built around the massive gathering and meticulous analysis of numerical data", with Microsoft managers expected to “crunch numbers for everything before they make decisions” (Smith 2015, p. 148). Likewise, Bloomberg has repeatedly stressed that his business philosophy was all about “following the data'', famously stating that: “In God we trust; everyone else bring data” (Bloomberg Philanthropies, 2013, p. 3). It is a philosophy that he brought with him when he moved to City Hall where, inspired by New Public Management ideals of transparency and accountability, he set up multiple systems to monitor and assess the efficiency of New York City's public services (Brash, 2011). Given their commitment to data and audit practices, it is perhaps no surprise that both Gates and Bloomberg saw the metrics and surveillance systems developed by epidemiologists as the perfect tool to monitor and assess their charitable efforts in global health. As Gates argued, “the metric of success [in global health] is lives saved': like 'units sold' and 'profits achieved', it is 'all very measurable and you can set ambitious goals and see how you do” (cited in Smith, 2015, p. 149). Bloomberg held similar views, as one epidemiologist working for him recounted:

Bloomberg is 100 per cent about data. His entire business is built on data. At City Hall he implemented all sorts of data-driven initiatives. This is a person very interested in data. Similarly, I am a trained epidemiologist and my focus is also on data … We both just want to know what impact we are having. That is why we fund measurement strategies like the GATS.

## Conclusion

Focusing on the Bloomberg Initiative, one of the largest international efforts to tackle the NCD epidemic currently spreading across the global South, this article explored the ubiquity and growing significance of numbers in the field of global health. In these concluding remarks, I want to underline and discuss what, I believe, are two of the major insights that this article can offer to other scholars in the social sciences and humanities who are researching and writing about global health metrics.

The first of these insights is the notion of epidemiological reason, which builds on the idea of thought style articulated by Hacking and others. Of course, the notion of epidemiological reason which I introduced and outlined in the first part of this article stems exclusively from my research on the Bloomberg Initiative. But, given that many of the forms of knowledge and practices associated with global health are shared across the field, it is reasonable to assume that other global health initiatives are informed by styles of thinking which, to borrow from Wittgenstein (2009, p. 36), have strong “family resemblances” to the notion of epidemiological reason I explored here. In any case, this notion of epidemiological reason draws our analytical gaze to the wider logic that knits together, organises and shapes the metrics that saturate the field of global health. As I showed in the article, this logic combines a multiplicity of different elements, from the ethical imperative to save lives and the expert figure of the epidemiologist to the political category of the global and the social-scientific technique of the survey. These different elements do not need to be entirely consistent with each other. An excellent example is the way epidemiological reason brings together and combines the technique of the social survey, which was developed by ardent proponents of the welfare state like Doll, with committed capitalists like Bloomberg and Gates. The article also highlighted the critical role that modern epidemiology plays in the making of epidemiological reason as with the social survey or the figure of the epidemiologist and it is to be expected that other, somewhat related bodies of knowledge which we did not have the time and space to explore here like health economics and evidence-based medicine play a similar role (Reubi, 2013; Adams, 2016a). Finally, while epidemiological reason might well foreclose other ways of thinking and being, it is important to understand that it is also central to the production of many of the ideas and objects that circulate within the contemporary political and moral economy of global health – as when epidemiologists, surveys and maps come together to turn smoking into today's largest 'global' preventable epidemic.

The second insight is the history or historical ontology of global health metrics sketched in the second part of the article. Drawing on a long tradition of genealogical studies, this history emphasises the importance of ruptures and eschews the Whiggish tales of scientific progress or neoliberal doom that can be found in parts of the literature. In particular, and contrary to what some scholars have argued, the article showed that many key ideas and practices associated with global health metrics like the desire to save lives and the trust in numbers are not a recent phenomenon associated with neoliberal rule but the product of a rupture in political thought at the end of the early modern era associated with the birth of biopolitics and statistical thinking. This is why, as I illustrated, there are many important similarities between the way William Farr and his allies used vital statistics to campaign for, set targets and assess sanitation efforts in mid-nineteenth-century UK and the way Tom Frieden and his colleagues have drawn on epidemiological data on smoking to devise and manage the Bloomberg Initiative. It would, however, be a mistake to think that nothing has changed since Farr and the development of vital statistics. Indeed, as the article also showed, the very practices of quantification and the life being quantified have changed over time. Specifically, the quantification practices that permeate the Bloomberg Initiative and other global health efforts stem from the emergence of surveillance medicine and the concomitant shift from vital statistics, with their concern for the impact of insalubrious environments on human mortality, to the social survey, with its interest in the biological, social, economic and political aspects of unhealthy lifestyles. Furthermore, the population whose life is quantified together with the infrastructure supporting these quantification efforts has, they too, changed since the time of Farr. In particular, the idea of a world population and the involvement of transnational partnerships of state and non-state actors that characterise efforts like the Bloomberg Initiative are the product of the twenty-first century reorganisation of world health around the idea of globalisation and a breakaway from the notion of national population and the state-centric infrastructure typical of vital statistics.

In conclusion, I believe that these different insights show the tremendous value of adopting a genealogical approach when seeking to make sense of the nuances and complexities of the contemporary spirit of quantification. In our age of big data in which numbers are so ubiquitous, this is not just the case for global health, which is the object of the present article, but for most other spheres of political, economic and social life too.

## Footnotes

1 The long eighteenth century – generally understood to last from the Restoration in 1660 or the Glorious Revolution of 1688 to the Battle of Waterloo in 1815 or the Reform Act of 1832 – is an expression coined by British and other historians to capture and analyse long-term historical developments that go beyond the more standard calendar-based definition of the eighteenth century.

2 As Etienne Balibar (1978) has shown, the notion of ‘epistemological rupture’ can be traced back to the work of French philosopher of science Gaston Bachelard. The notion was later taken up and reworked by the French Marxist thinker Louis Althusser and both Georges Canguilhem and Michel Foucault before being adopted by Ian Hacking.

3 The name of the WHO’s annual world statistical report has changed over time. It was entitled the Annual Epidemiological and Vital Statistics up to 1964, when it was renamed the World Health Statistics Annual. It kept this title until 1995 and, after a ten-year absence, came back as World Health Statistics.
